# Mock-up performance evaluation study for crack reduction of blast furnace slag powder concrete mixed with expansive and swelling admixtures

**DOI:** 10.1038/s41598-024-52998-1

**Published:** 2024-01-29

**Authors:** Sanghyuck Yoon, Wonyoung Choi, Chansoo Jeon

**Affiliations:** 1 Department of Building Research, Korea Institute of Civil Engineering and Building Technology, Goyang, 10223 Korea; 2 Construction Test and Certification Department, Korea Institute of Civil Engineering and Building Technology, Goyang, 10223 Korea

**Keywords:** Engineering, Materials science

## Abstract

In this study, the drying shrinkage and crack reduction characteristics of blast furnace slag concrete mixed with expansive and swelling admixtures were investigated. Basic performance experiments were conducted using different mixtures of ground granulated blast-furnace slag (GGBS), with calcium sulfoaluminate as the expansive admixture and bentonite and hydroxypropyl methyl cellulose (HPMC) as swelling admixtures. Bentonite outperformed HPMC as a swelling admixture. Specimens were then prepared for mock-up tests to evaluate the drying shrinkage of blast furnace slag concrete with different combinations of bentonite, a hardening accelerator, and a self-healing agent. The addition of bentonite and calcium phosphate as a self-healing agent in small quantities reduced the drying shrinkage of the specimens, thereby reducing cracks. The cement mixture composed of 30% GGBS, 1% bentonite, and 1% calcium phosphate (30-E1-I1) showed the optimal performance among the specimens. Further, crack monitoring was performed in concrete made with ordinary Portland cement and optimal mixture 30-E1-I1. No cracks were observed for the optimal mixture. This shows that GGBS concrete can be used in practical and field applications, subject to mid- and long-term tests for cracking.

## Introduction

Among industrial byproducts that are suitable for use as admixtures, the utilization of ground granulated blast-furnace slag (GGBS) has increased recently. Admixtures are used in the production of specific types of concrete owing to their economic feasibility, stability, and eco-friendliness^1^. KS F 2563 categorizes concrete into types 1, 2, and 3 depending on its specific surface area and activity index. Type 3 concrete, which has a specific surface area of 4,000–6,000 cm^2^/g, is the most predominantly used^2^. To improve the initial strength using GGBS, a method of increasing the latent hydraulic reaction by increasing the surface area through fine powder has been presented. However, this method is only used in special cases because increasing fineness increases production costs, which may have an adverse effect on economic feasibility.

A glossy oxide film is formed on the surface of the GGBS. Thus, hydration cannot occur without an alkali stimulus. Because the overall surface area increases as the powder becomes finer, hydration activity may be accelerated if alkali activators are used. After the reaction of GGBS with water, the elution of Ca^2^ + ions forms a glass film on the surface of GGBS, which inhibits hydration. Alkali activators may be used to remove this film, thus improving hydration^3^.

It has been reported that concrete mixed with GGBS has low hydration heat and significantly improved watertightness, long-term strength, alkali-aggregate reaction, freeze–thaw, and chemical corrosion. However, problems with the early strength, autogenous shrinkage, and initial drying shrinkage persist. In particular, concrete containing GGBS generates a small amount of calcium hydroxide owing to its low cement content, and carbonation resistance decreases depending on the curing and mixing conditions^4^.

Existing studies have been undertaken to address the aforementioned problem. One notable endeavor involved an experimental study employing a ring test, aimed at mitigating shrinkage cracks by substituting GGBS for fine aggregate weight. The objective was to enhance flexural strength, compressive strength, and modulus of elasticity^5^. Furthermore, research has focused on the development of a predictive method for concrete cracks, leveraging ring test results in conjunction with a network-based system and machine learning algorithms. This methodology has been applied to estimate cracks by utilizing digital images on actual construction sites^6–8^. Collectively, these initiatives underscore the ongoing progress in technology for enhancing the crack characteristics of concrete through the incorporation of GGBS.

In addition, from a material perspective, it is necessary to conduct research on improving the cracking characteristics of GGBS concrete using a small amount of additives. Recently, the construction standards of Korea have been revised to consider concrete cracks smaller than 0.3 mm as defects if they result in leakage or are located in steel reinforcement. In addition, microcracks near plastered and painted areas and crazing that causes aesthetic problems are considered as defects. The material approach to concrete crack control can be divided into expansive admixture utilization technology, fiber reinforcement methods, and maintenance technology using the injection method after cracking. Expansive admixture utilization technology involves securing the amount of shrinkage that may occur from the initial curing process until atmospheric drying considering expansibility; admixtures such as expansive cement and calcium sulfo-aluminate (CSA) are used to remedy this. However, concrete cracking may occur due to excessive material expansion and the curing process. Therefore, it is necessary to inhibit the movement of moisture and air by adjusting the total number and size of pores in concrete, thus controlling the characteristics that can prevent cracking during drying shrinkage by securing a stable pore structure. Materials or technology, such as alkali activators, that can improve characteristics such as concrete watertightness, initial strength, and crack growth retardation with an increase in the substitution amount of slag cement, are required.

We previously conducted a study on the plasticity and drying shrinkage crack control of cement mixed with a CSA-based expansive admixture and moisture-absorbent swelling admixture to improve the initial strength degradation caused by the use of GGBS and confirmed their effectiveness for concrete production by controlling the GGBS content, influence of expansive admixtures, and mix proportions^9^.

In this study, basic performance experiments and mock-up tests were conducted on blast furnace slag concrete with a fineness of 4,000 cm^2^/g, a GGBS substitution rate of 30%, and expansive and swelling admixtures, which is the most commonly used combination in the ready-mixed concrete industry, to quantitatively investigate drying shrinkage and examine field applicability. In addition, restrained drying shrinkage crack tests conducted using underground parking lot slabs were evaluated. Furthermore, considering economic feasibility, a minimal amount of hardening accelerator and self-healing agent were used to investigate the influence of adding an admixture on slag properties, thereby presenting basic data for reducing cracks in blast furnace slag concrete to overcome the limitation of early strength degradation.

## Basic characteristics experiment

Basic characteristics of concrete were assessed for verification purposes by performing laboratory experiments on five samples containing expansive and swelling admixtures.

### Test plan

#### Test plan and method

Tests were conducted based on a compressive strength of 30 MPa, which is the normal range of application of concrete strength, and a GGBS substitution rate of 30%. In all the samples, the content of the expansive admixture (CSA) was within 5%. Na + -type bentonite swelling admixture 1% (E1) or hydroxypropyl methyl cellulose swelling admixture 1% (E2) was added as the swelling admixture depending on the sample, and 0.2% of lithium carbonate was selected as the organic salt-based hardening accelerator. Lithium carbonate facilitates the hydration of C3A and actively contributes to the formation of ettringite(3CaO·Al_2_O_3_·3CaO·3SO_3_·32H_2_O). This process results in rapid hardening, consequently enhancing the initial strength^10^.

To analyze the hardness of the precipitate, self-healing calcium phosphate was mixed at 1% (I1) and 3% (I3) for comparison. The fine and coarse aggregates used in this study were converted based on the fine aggregate ratio and unit weight of cement, and the field experiment was conducted by adjusting the unit water content and the amount of superplasticizer. The experimental design parameters are listed in Table 1, and the concrete mix proportions are listed in Table 2.

**Table 1:** Experimental design and factors.

Division	Materials	Factor	Type of experiment
Concrete design strength		30 MPa	・Air content ・Slump ・Compressive strength ・Length change ・Crack degree
Binder	GGBS: OPC	30: 70	
Expandable material	Calcium sulfo-aluminate (CSA) expansive admixture	0%, 5%	
Swelling material	Na + -type bentonite swelling admixture	0%, 1%	
Hardening accelerator	Lithium carbonate (Li2CO3) hardener	0%, 0.2%	
Self-healing agent	Self-healing calcium phosphate	0%, 1%, 3%

**Table 2:** Concrete mix proportions.

Symbol	W/B (%)	S/a (%)	W (kg/m^3^)	Material quantity per unit volume (kg/m^3^)									Retarder (%)	AE agent (%)	SP agent (%)
				GGBS	OPC	CSA	E1	E2	H	I	G	S			
30-OPC	40	48.6	160	120	280	0	0	0	0	0	890	840	0.2	0.009	0.8
30-E1-I1	40	48.6	160	120	260	20	4.0	0	0.8	4.0	890	840	0.2	0.009	0.8
30-E1-I3	40	48.6	160	120	260	20	4.0	0	0.8	12.0	890	840	0.2	0.009	0.8
30-E2-I1	40	48.6	160	120	260	20	0	4.0	0.8	4.0	890	840	0.2	0.009	0.8
30-E2-I3	40	48.6	160	120	260	20	0	4.0	0.8	12.0	890	840	0.2	0.009	0.8

GGBS: Ground granulated blast-furnace slag.

OPC: Ordinary Portland cement.

CSA: Calcium sulfo-aluminate (CSA) expansive admixture.

E1: Na + -type bentonite swelling admixture.

E2: Hydroxypropyl methyl cellulose swelling admixture.

H: Lithium carbonate (Li2CO3) hardener.

I: Self-healing calcium phosphate.

Slump and air content tests were conducted in accordance with the KS F 2402 test method for concrete slump and KS F 2421 test method for air content of fresh concrete using the pressure method, and the target slump and air content were set to 180 ± 25 mm and 4.5 ± 1.5%, respectively, which are standards for ordinary concrete, in accordance with KS F 4009 for ready-mixed concrete. In addition, for hardened concrete, the compressive strength was measured at 3, 7, and 28 d of age in accordance with the KS F 2405 test method for compressive strength of concrete.

#### Materials

Cement: For the blast furnace slag concrete test, OPC with a fineness of 3,483 cm^2^/g produced in Korea was used as shown in Tables 3 and 4.
(2) GGBS: In addition to cement, GGBS with a fineness of 4,650 cm^2^/g was used for the watertightness of the pore structure as shown in Table 5. In particular, a high-quality material with an ignition loss of 0.9% and almost no foreign matter was used.
Expansive admixture: The expansive admixture used was synthesized by Company I in Korea using CSA-based bauxite from China. It had a fineness of 3,811 cm^2^/g, density of 2.89 g/cm^3^, and weight that was slightly lower than that of cement but similar to that of GGBS as shown in Table 6.
Swelling admixture: Na + -type bentonite and fiber-type hydroxypropyl methylcellulose (HPMC) in powder form were used as the main components as shown in Table 7.
Aggregate: River sand and 25 mm crushed coarse aggregate from Oksan, Cheongwon-gun, and Chungbuk-do, Korea, were used as fine and coarse aggregates, respectively as shown in Table 8.
Chemical admixture: A naphthalene-based high-performance water-reducing agent suitable for “KS F 2560 chemical admixture for concrete” was used as the chemical admixture, and high-quality alcohol was used as the AE agent as shown in Table 9.

**Table 3:** Physical properties of cement.

Density (g/cm^3^)	Fineness (cm^2^/g)	Setting time (min)		Compressive strength (MPa)		
		Initial set	Final set	3 d	7 d	28 d
3.15	3,483	208	351	20.4	29.4	`38.7

**Table 4:** Chemical properties of cement.

Division	CaO	SiO_2_	Al_2_O_3_	MgO	Fe_2_O_3_	SO_3_	K_2_O	Na_2_O
Content (%)	62.2	20.3	6.0	3.39	3.2	2.4	0.78	0.14

**Table 5:** Properties of GGBS.

Density (g/cm^3^)	Fineness (cm^2^/g)	Basicity	Chemical composition (%)						
			SiO_2_	Al_2_O_3_	Fe_2_O_3_	CaO	MgO	SO_3_	ig.loss
2.931	4,650	1.9	31.2	14.1	0.3	44.8	6.2	2.5	0.9

**Table 6:** Physical and chemical properties of CSA-based expansive admixture.

Density (g/cm^3^)	Fineness (cm^2^/g)	Chemical composition (%)						
		ig.loss	SiO_2_	Al_2_O_3_	Fe_2_O_3_	CaO	MgO	SO_3_
2.89	3,811	0.1	3.7	9.0	1.5	55.7	1.6	28.4

**Table 7:** Physical properties of swelling material.

Category	Main component	Form	Color	Viscosity
Bentonite	Na +	Powder	Yellow	1,000 cps
HPMC	Cellulose	Powder	White	1,500 cps

**Table 8:** Aggregate quality characteristics.

Category	Density (g/cm^3^)	Fineness modulus	Water absorption (%)	Mass per unit volume (kg/m^3^)	Percentage passing through a 0.08 mm sieve (%)
Fine aggregate	2.51	2.90	0.46	1,490	0.30
Coarse aggregate	2.63	7.04	0.58	1,532	0.40

**Table 9:** Physical properties of admixtures.

Category	Main component	Form	Color	Density (g/cm^3^)
Hardener	Lithium carbonate	Powder	White	–
Self-healing calcium phosphate	Cl (calcium phosphate)	Powder	White	–
Retarder	Fluorine-based	Powder	yellow	–
High-performance water-reducing agent	Naphthalene	Liquid	Light brown	1.21
AE agent	High-quality alcohol	Liquid	Pale yellow	1.01

### Result

#### Slump & air content

**Figure 1:**
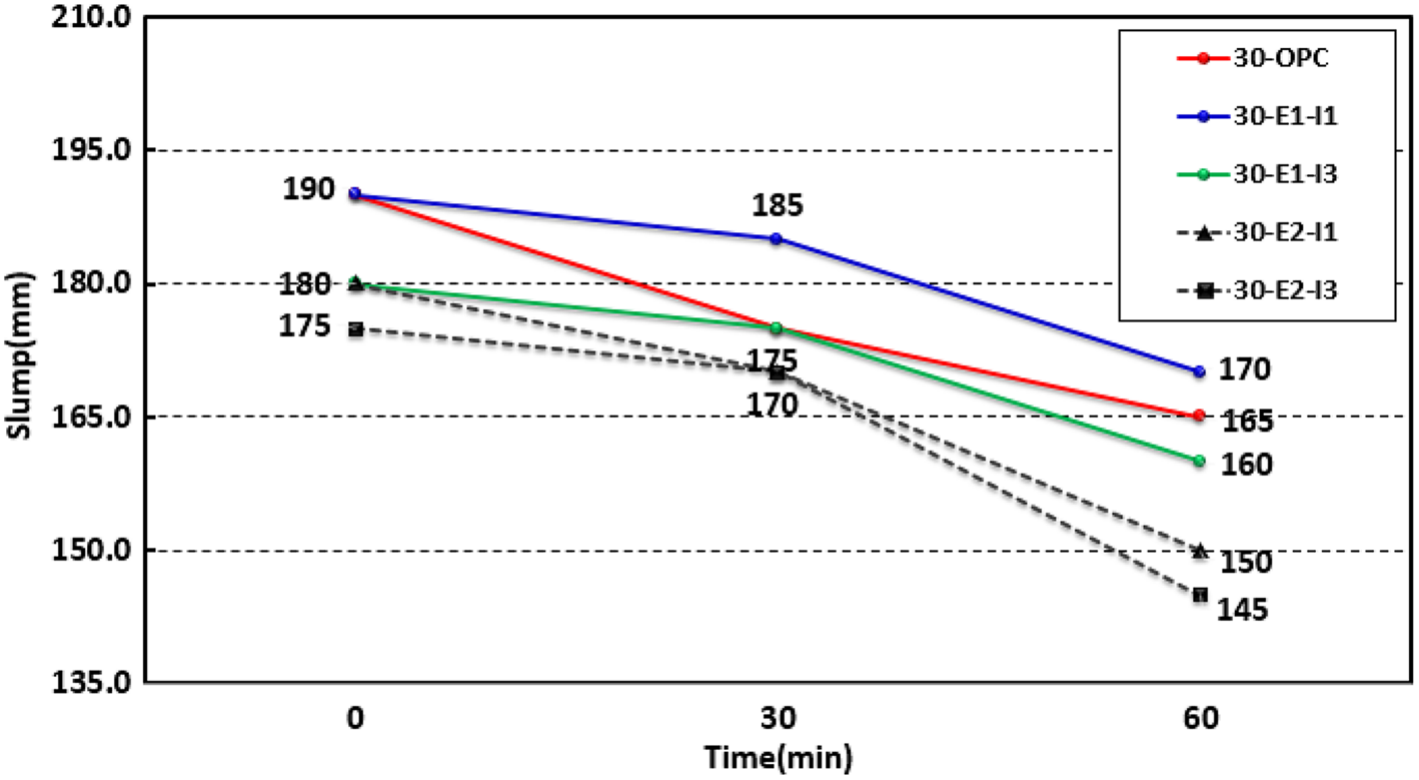
Slump test results.

**Figure 2:**
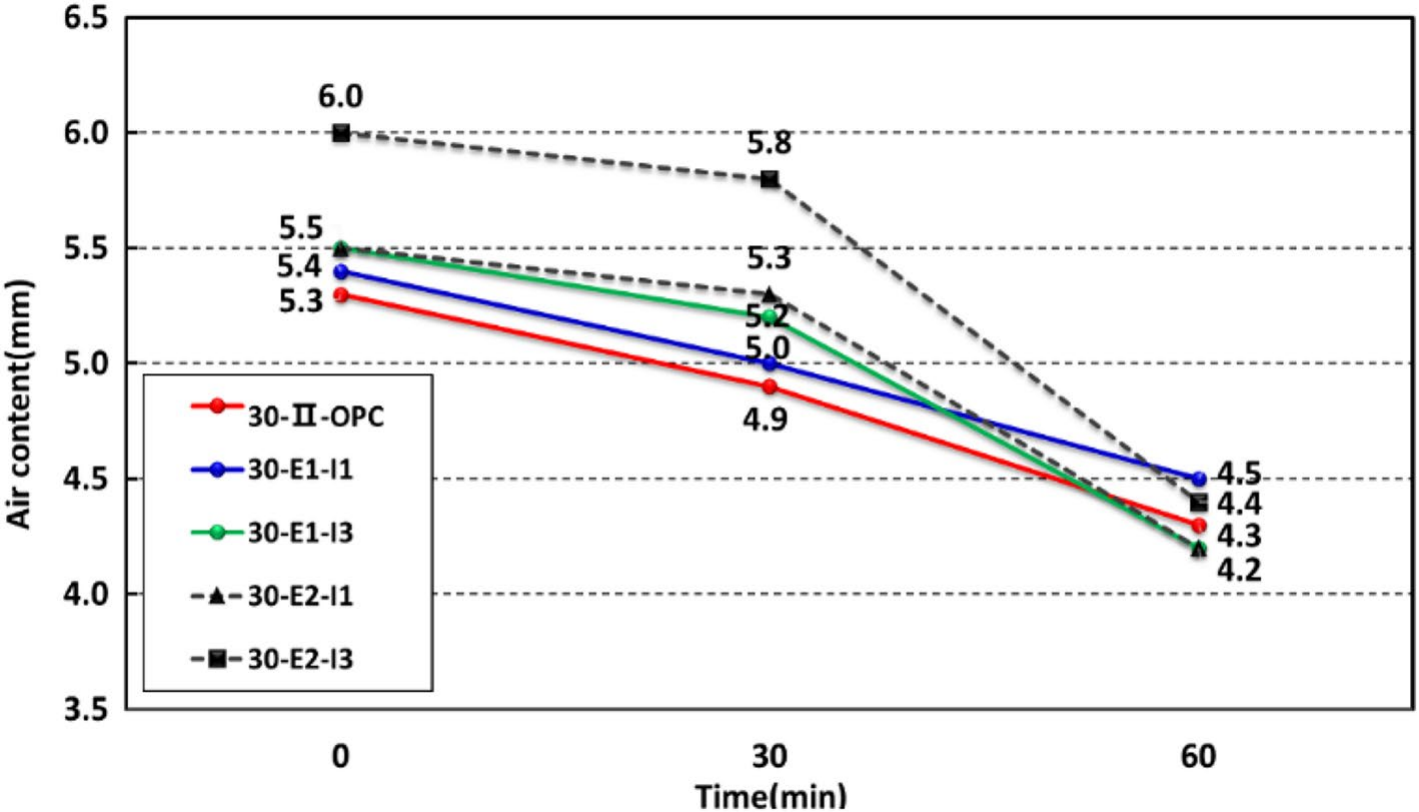
Air content test results.

Figures 1 and 2 show the slump and air content, respectively, of concrete before hardening. The slump values at 0, 30, and 60 min were 190, 175, and 165 mm, respectively, for 30-OPC; 190, 185, and 170 mm, respectively, for 30-E1-I1; 180, 175, and 160 mm, respectively, for 30-E1-I3; 180, 170, and 150 mm, respectively, for 30-E2-I1; and 175, 170, and 145 mm, respectively, for 30-E2-I3. Fluidity for 30-OPC, 30-E1-I1, and 30-E1-I3 was achieved by meeting the target slump of 180 ± 25 mm in the “KS F 4009 ready-mixed concrete” standard, whereas fluidity for 30-E2-I1 and 30-E2-I3 could not be achieved because their slumps were lower than those of other mixes and could not meet the standard.

In addition, compared to the 0–30 min period, the slump values of the other mixes decreased more rapidly in the 30–60 min time period than those of 30-OPC. This is attributed to the accelerated change over time in the concrete sample for which expansive and swelling admixtures were used. A swelling admixture with a high moisture retention capacity may cause workability problems in its fresh state owing to rapid water absorption. To address this problem, it is necessary to partially use a retarder and limit the use of a swelling admixture^11^. In addition, because the change in characteristics over time rapidly deteriorate in high-strength concrete mixes with high contents of expansive and swelling admixtures, securing workability using superplasticizers and retarders would be beneficial^12^.

The air content measurements over time satisfied the criterion of 4.5 ± 1.5%. The results were found to be 5.3, 4.9, and 4.3% for 30-OPC; 5.4, 5.0, and 4.5% for 30-E1-I1; 5.5, 5.2, and 4.2% for 30-E1-I3; 5.5, 5.3, and 4.2% for 30-E2-I1; and 6.0, 5.8, and 4.4% for 30-E2-I3. However, there was a reduction in air content due to the increased use of swelling admixture and the use of E2, indicating the need to control the unit water content and the amount of AE agent in the future^13^.

Additionally, prior studies have reported that the high-performance water-reducing agent, functioning as an air-entraining agent, demonstrates remarkable efficacy when used in combination with an AE agent as an auxiliary agent to attain the required air volume^14^. Moreover, it has been noted that the air content in the high-performance water reducer gradually decreases immediately after mixing, both before and after addition, depending on the concrete temperature. The experimental results revealed a similar trend, attributed to the chemical reactions involving high-performance water reducers and AE agents^15^.

#### Compressive strength

**Figure 3:**
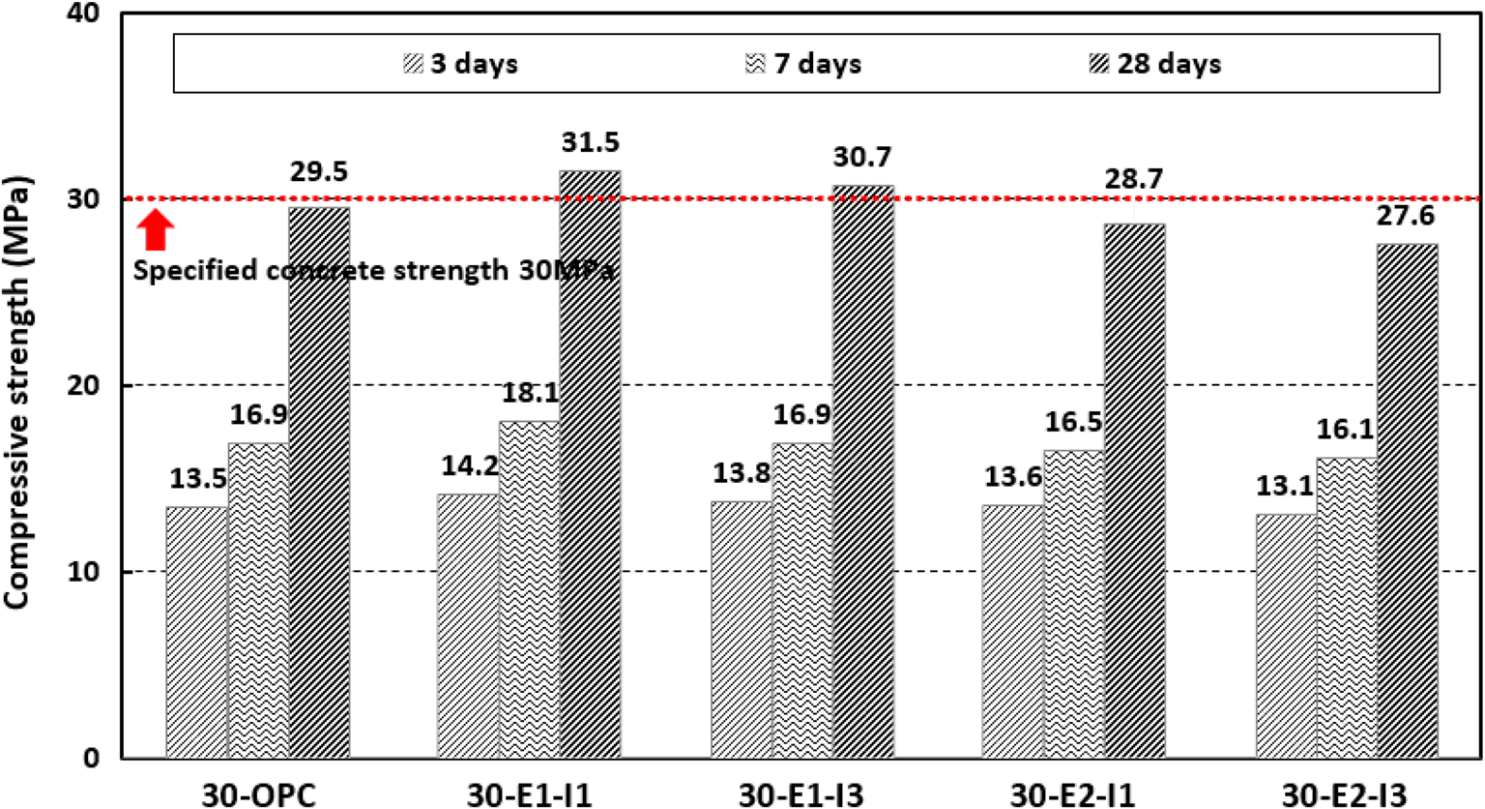
Compressive strength test results.

Figure 3 shows the compressive strength test results at 3, 7, and 28 d. The compressive strength tended to increase with increasing age, with values of 13.1–14.2 MPa, 16.1–18.1 MPa, and 27.6–31.5 MPa at ages of 3, 7, and 28 d, respectively. At 28 d, 30-E1-I1 and 30-E1-I3, that is, the concrete for which bentonite-based swelling admixture was used, exhibited an increase of 100% in strength, indicating that they were more favorable for strength development than concrete mixed with OPC and HPMC-based swelling admixtures. This appears to be because the concrete for which a bentonite-based swelling admixture was used formed a more watertight structure during the mixing step by securing the air content and slump, as in the results of the fresh concrete experiment.

Based on the above results, it was deduced that practical verification is necessary through field application tests for 30-OPC, 30-E1-I1, and 30-E1-I3, that is, concrete mixed with ordinary cement and concrete mixed with GGBS, expansive admixture, and bentonite-based swelling admixture.

## Mock-up test

### Plan and method

#### Production of mock-up test specimens

Based on the results of the basic performance experiments, the characteristics of slag concrete containing expansive and swelling admixtures were analyzed and the drying shrinkage was quantitatively investigated through the mock-up test.

**Figure 4:**
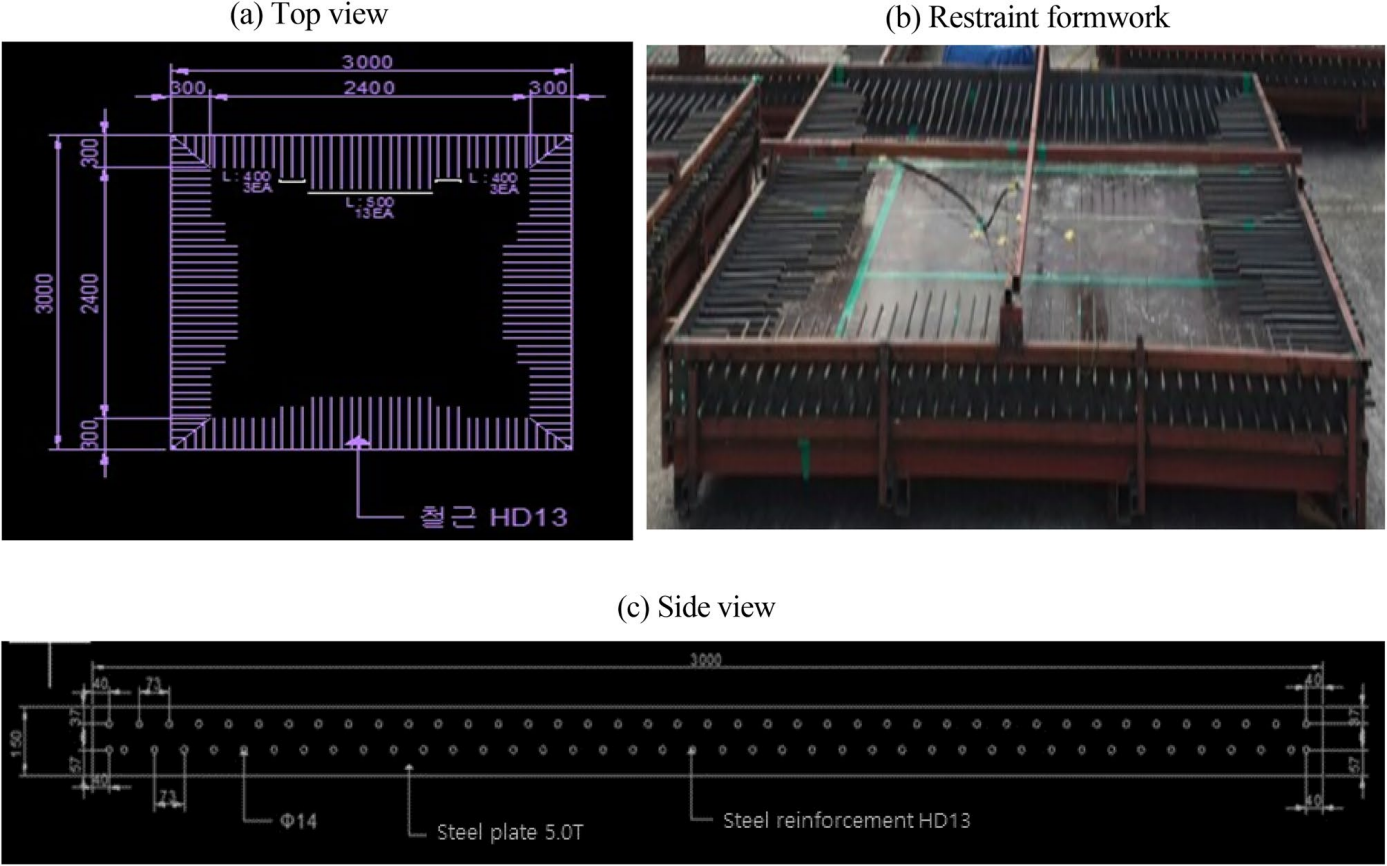
Mock-up test specimen.

**Figure 5:**
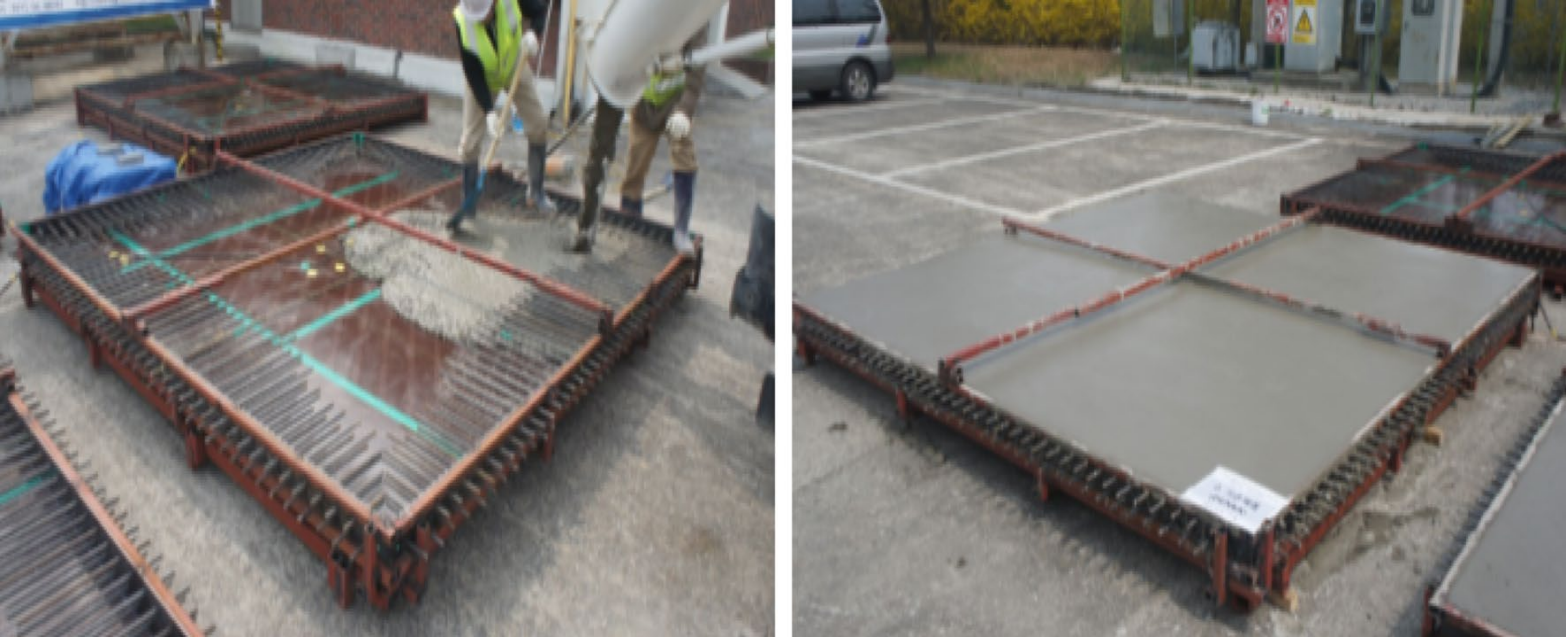
Mock-up specimen casting.

The strength setting of concrete structures can be approached in various ways; however, the mock-up test was conducted on 30 MPa concrete, which is the normal strength concrete application range. Based on the E1 mix that used 1% swelling admixture, restraint formwork was selected as the crack restraint, and a size of 3 m (width) × 3 m (length) × 0.15 m (height) was selected. Ready-mixed concrete was poured under the mix conditions of 30-OPC, 30-E1-I1, and 30-E1-I3. Figures 4 and 5 show the mock-up test specimen and casting, respectively.

#### Method

To evaluate the flow characteristics of fresh concrete, ready-mixed concrete samples were collected and fluidity tests were conducted, including the air content and slump flow.

After ready-mixed concrete production, the compressive strength specimens were prepared at the site in accordance with KS F 2403. After arriving at the site, curing was performed using the standard water curing method. The compressive strength was evaluated in accordance with KS F 2405 at 3, 7, and 28 d, and the average value of three specimens was adopted as the test result^16^.

Because concrete with a low W/B ratio and a high binder content per unit volume of concrete exhibits an increase in autogenous shrinkage, the length change characteristics were examined and analyzed to investigate the characteristics of autogenous shrinkage cracking^17^. A strain gauge was embedded horizontally in the center of the specimen at a distance of 60 mm from the bottom as a length change sensor, and measurements were performed hourly using a data logger.

For the crack monitoring of concrete mixed with GGBS and a crack-reducing agent, the number of cracks, crack width, and crack length were examined after 5 days, and the results were compared with the allowable crack width in the crack defect decision criteria for concrete structures.

### Results

#### Slump & Air content

**Figure 6:**
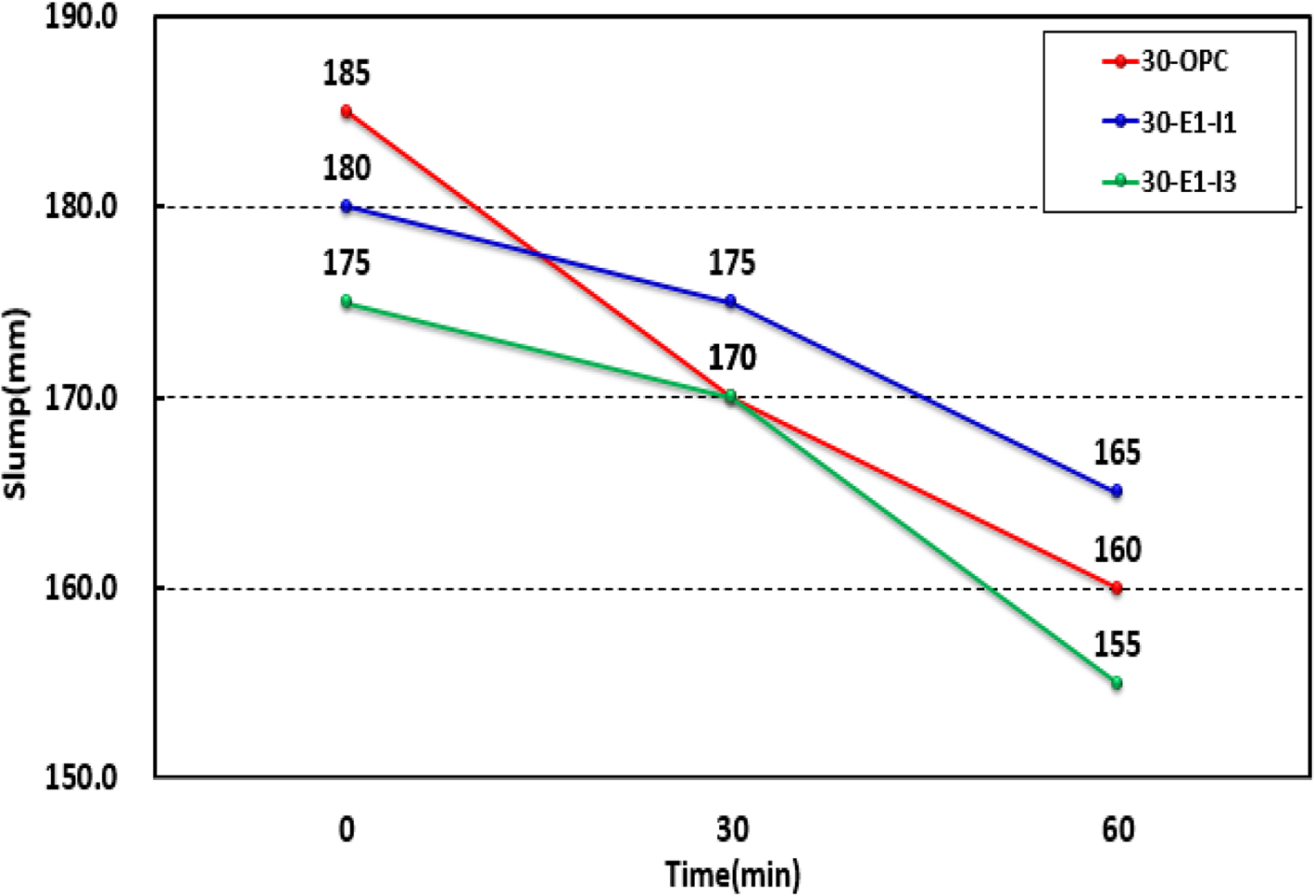
Slump test results for the mock-up.

As shown in Fig. 6, the slump values of each mock-up test specimen at 0, 30, and 60 min were 185, 170, and 160 mm, respectively, for 30-OPC; 180, 175, and 165 mm, respectively, for 30-E1-I1; and 175, 170, and 155 mm, respectively, for 30-E1-I3. The slump after 60 min decreased in the order: 30-E1-I1, 30-OPC, and 30-E1-I3; however, the concrete fluidity was not significantly affected because the results met the target slump of 180 ± 25 mm in accordance with KS F 4009.

**Figure 7:**
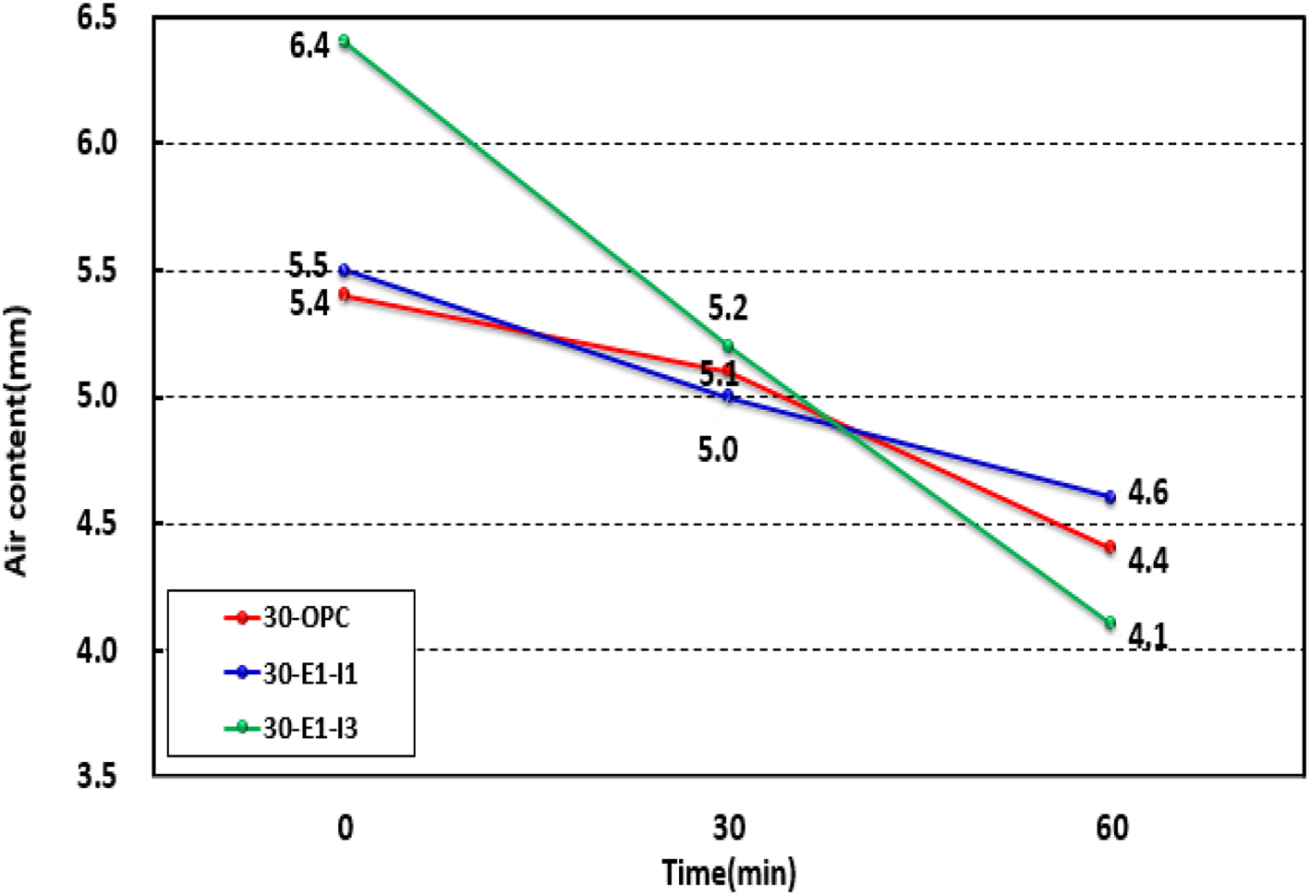
Air content test results for the mock-up.

Figure 7 shows the air content of each mock-up test specimen at 0, 30, and 60 min. All of the results met the standard and ranged between 4.4 and 5.4%, 4.6–5.5%, and 4.1–6.4% for 30-OPC, 30-E1-I1, and 30-E1-I3, respectively. At 30 and 60 min, the results decreased by 0.3–1.0% for 30-OPC, 0.5–0.9% for 30-E1-I1, and 1.2–2.3% for 30-E1-I3 compared to those at 0 min, indicating that controlling the unit water content and amount of the AE agent is essential for the application of concrete mixed with GGBS and a crack-reducing agent. Moreover, reports indicate that the air volume associated with the high-performance water-reducing agent, serving as an air-entraining agent, gradually decreases immediately after mixing, both before and after addition. A parallel trend was observed, believed to be attributed to the chemical reaction between the high-performance water-reducing agent and the AE agent^18,19^.

#### Compressive strength

**Figure 8:**
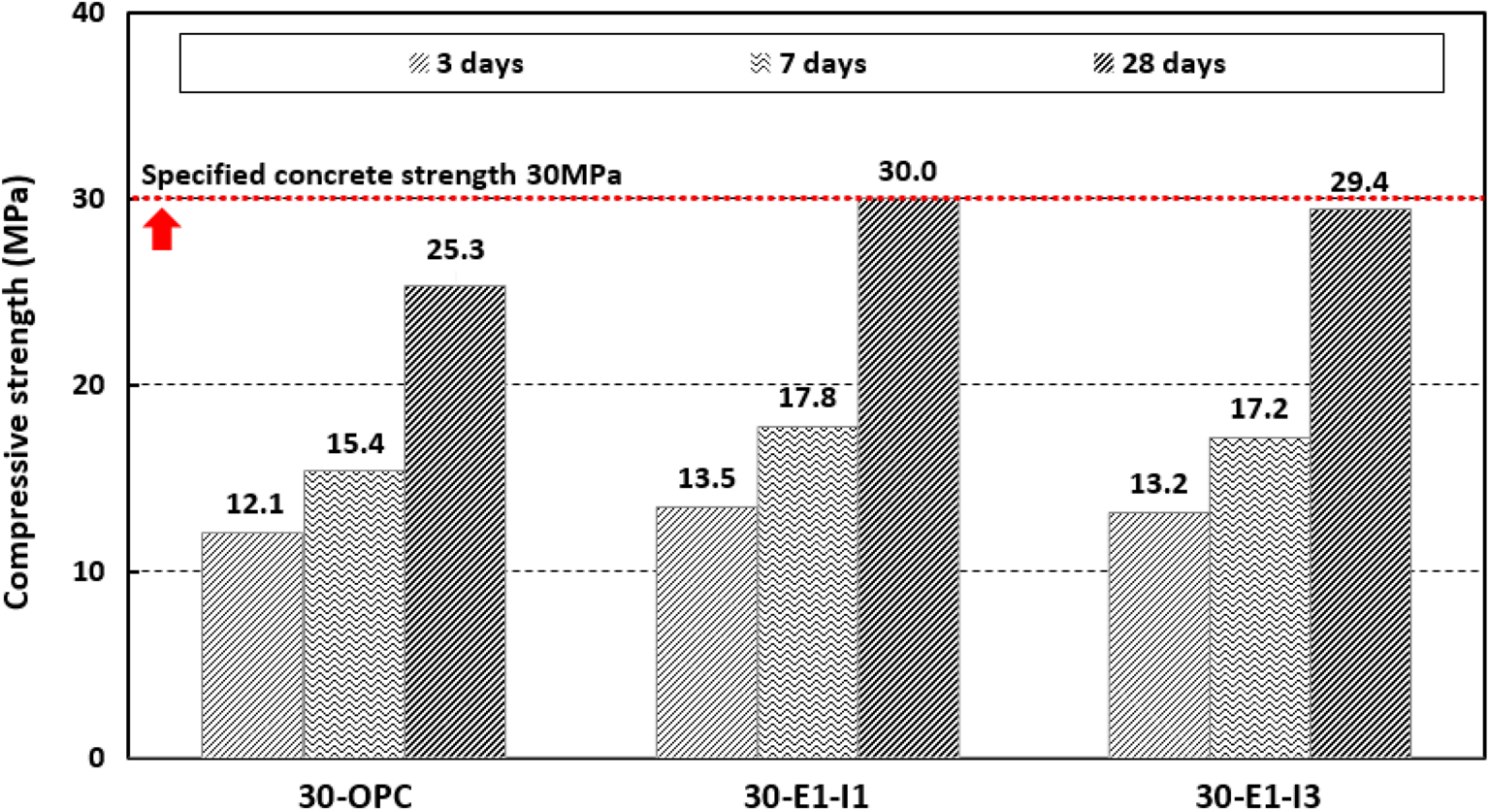
Compressive strength test results for mock-up.

Figure 8 shows the compressive strength results at 3, 7, and 28 d. The results were 12.1–25.3 MPa for 30-OPC, 13.5–30.0 MPa for 30-E1-I1, and 13.2–29.4 MPa for 30-E1-I3. At 28 d, the strengths of 30-E1-I1 and 30-E1-I3 were approximately 18.6% and 16.2% higher than that of 30-OPC. According to a previous study, using calcium phosphate with cement increases hardness by generating apatite as a hydration product^20^. In this study, calcium phosphate was used as the minimal admixture, and 30-E1-I1 with 1% calcium phosphate outperformed 30-E1-I3 with 3% calcium phosphate, indicating that a more efficient self-healing effect can be achieved by adding calcium phosphate in small quantities.

### Length change

**Figure 9:**
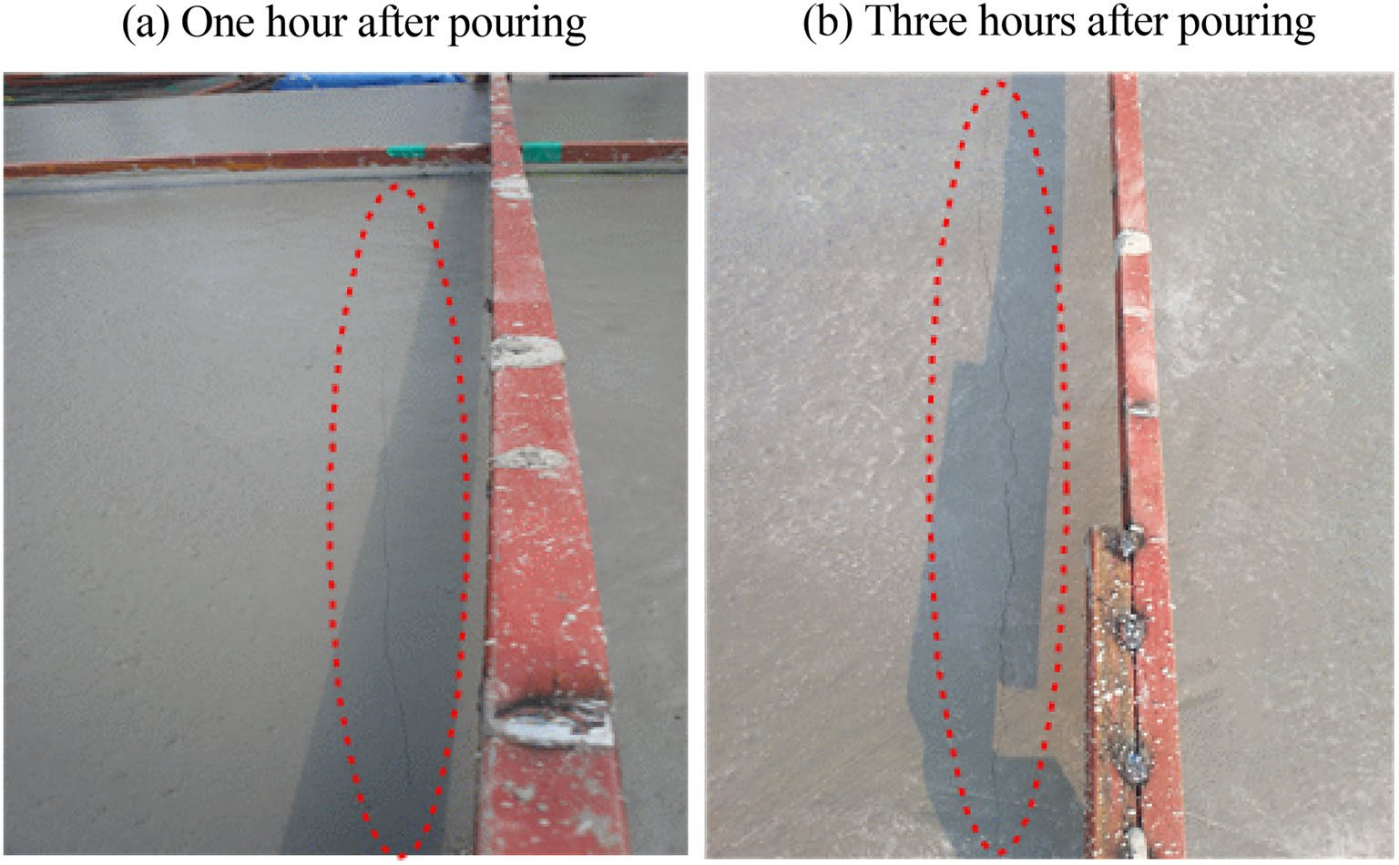
Initial crack after mock-up casting (30-OPC).

**Figure 10:**
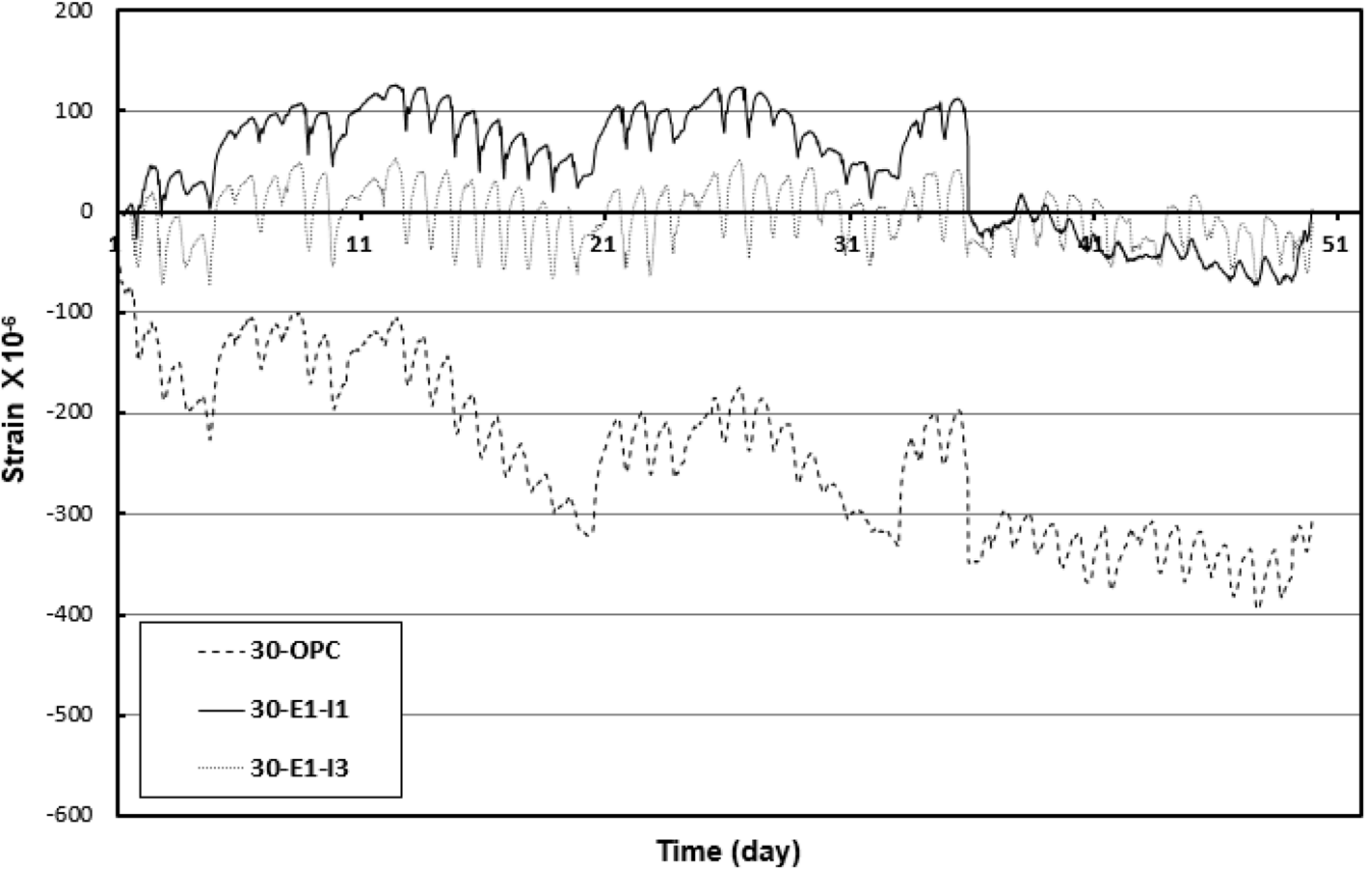
Mock-up length change test results.

Figure 9 shows the initial crack after mock-up casting. The test results are shown in Fig. 10. The measurement results appeared in the form of a curve, indicating that they were affected by temperature and humidity changes at the site at the time of measurement. Overall, the results were -390–200 × 10^−6^ for 30-OPC, –90–110 × 10^−6^ for 30-E1-I1, and − 86–50 × 10^−6^ for 30-E1-I3. Compared to 30-OPC, 30-E1-I1 and 30-E1-I3 exhibited a drying shrinkage reduction rate of approximately 25–50% and 15–25%, respectively, confirming that the length change decreased in the following order: 30-OPC, 30-E1-I3, and 30-E1-I1.

In the case of 30-OPC, rapid drying shrinkage occurred from the beginning, and shrinkage of approximately − 147 × 10^−6^ occurred over one day. Subsequently, drying shrinkage occurred continuously until approximately − 390 × 10^−6^, indicating a high probability of cracking.

In the case of 30-E1-I1 and 30-E1-I3, which used an expansive admixture, there was a large initial expansion, followed by relatively small shrinkage. This indicates that the use of an expansive admixture provides initial expansibility and inhibits shrinkage by increasing the density of the microstructure through continuous hydration of the internal expandable material^21^.

In general, tensile stress is generated by hydration heat that occurs during the drying shrinkage or hardening of concrete, and cracking occurs when the stress exceeds the tensile strength of the concrete. From this perspective, the effect of an expansive admixture is one of the methods for reducing cracks by increasing tensile strength^22^. As shown in Fig. 10, 30-E1-I1 mixed with 1% of calcium phosphate showed a higher expansion effect than 30-E1-I3 mixed with 3% calcium phosphate. This is because the increase in calcium phosphate content decreased the expansion effect by inhibiting the hydration reaction caused by the generation of ettringite and calcium hydroxide by the expansive admixture.

### Crack monitoring

**Figure 11:**
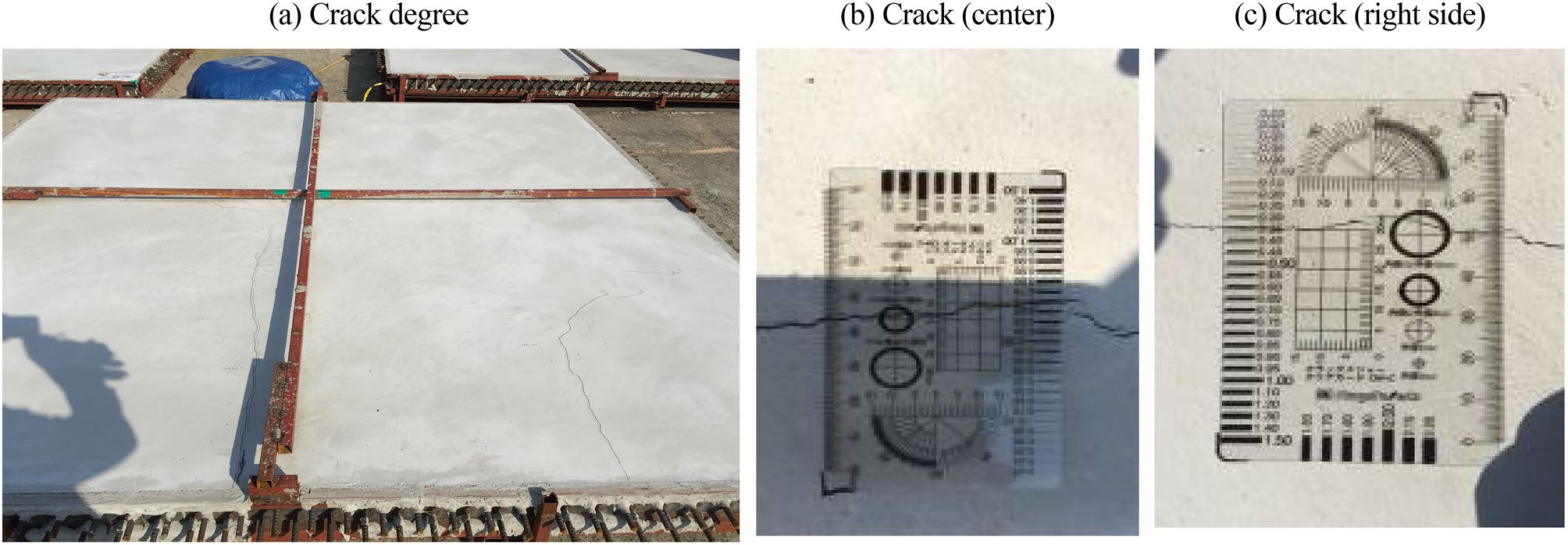
Crack measurement after curing (30-OPC).

**Figure 12:**
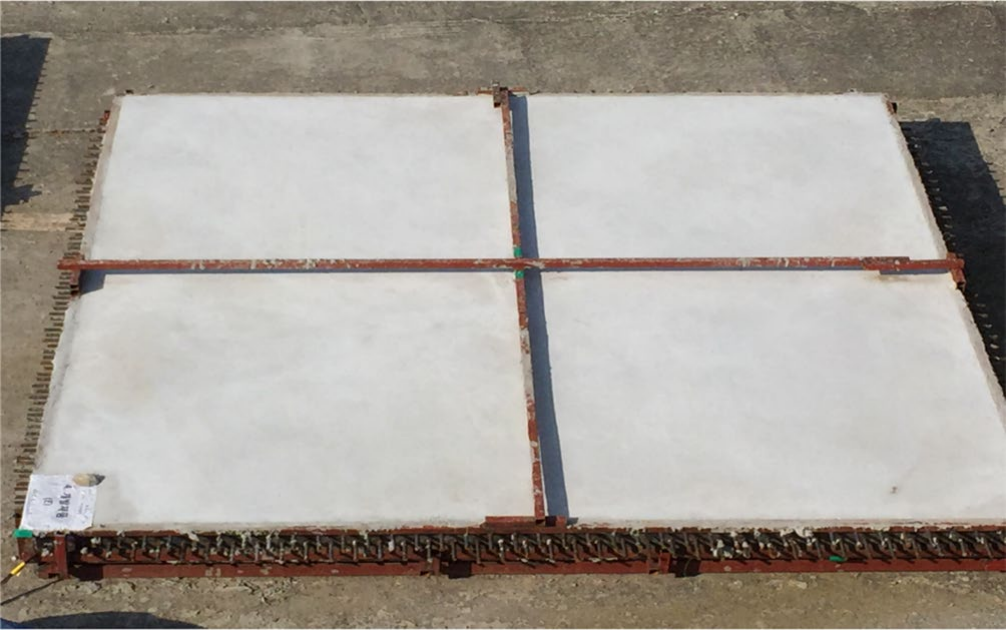
Crack measurement after curing (30-E1-I1).

**Figure 13:**
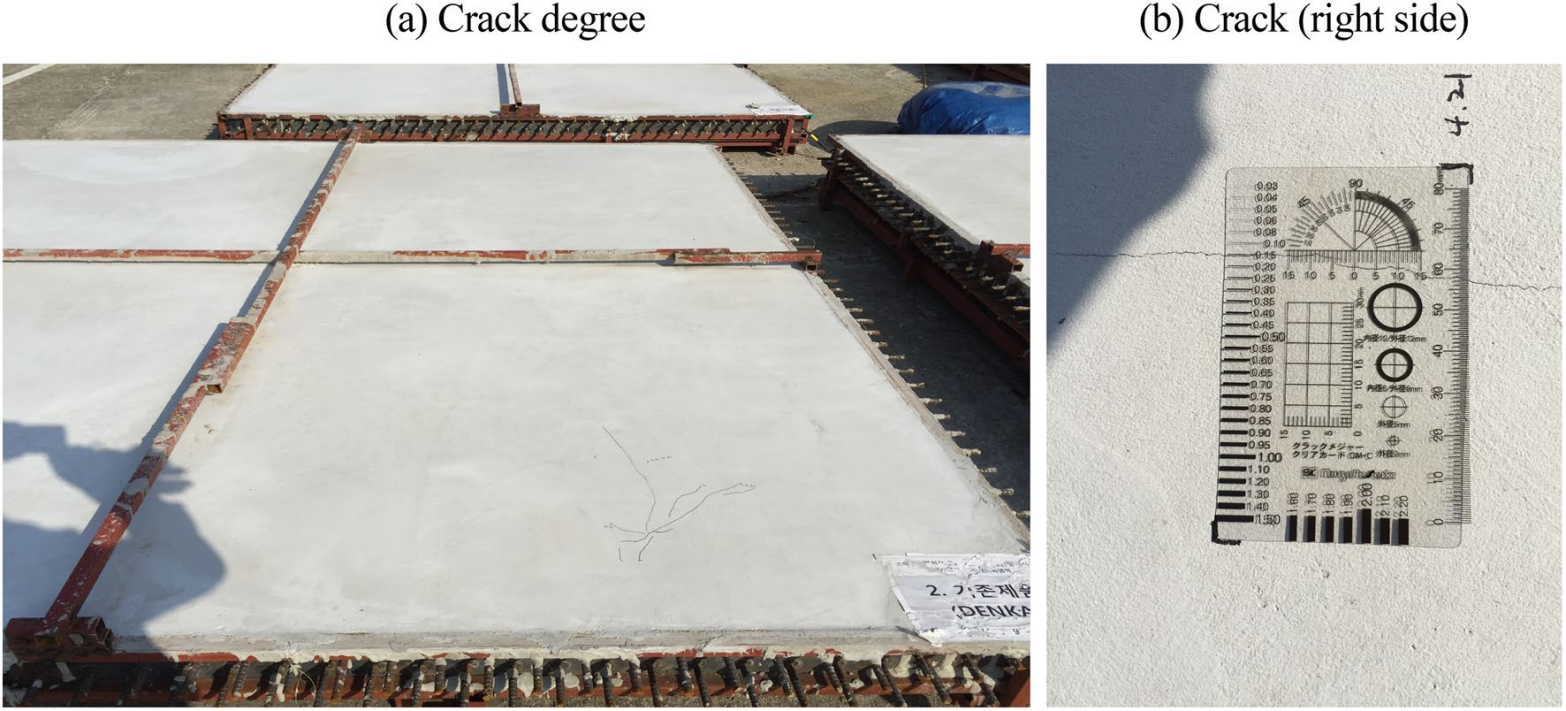
Crack measurement after curing (30-E1-I3).

Tables 10 and 11 lists the crack monitoring results for the mock-up test specimens at 50 d. As shown in Figs. 11, 12, 13, the crack width and length were found to be 0.3–0.7 mm and 7.0 m, respectively, for 30-OPC; 0.0 mm and 0.0 m, respectively, for 30-E1-I1; and 0.05–0.15 mm and 0.5 m, respectively, for 30-E1-13. Cracking occurred 4 h after concrete pouring for 30-OPC and 14 d after concrete pouring for 30-E1-I3. 30-E1-I1 exhibited no crack and met the allowable crack width of 0.3 mm in the crack defect criteria for concrete structures^23^.

**Table 10:** Crack monitoring results.

Division	Number of cracks (EA)	Crack length (m)	Crack width (mm)
30-OPC	6	7.0	0.3 ~ 0.7
30-E1-I1	0	0	0
30-E1-I3	2 + delusion crack	0.5	0.05 ~ 0.15

**Table 11:** Crack width of test specimen.

Division	Review of concrete crack defect criteria (allowable crack width)	
	0.3 mm or less (acceptable)	0.3 mm or more (unsuitable)
30-OPC	occurrence	Occurrence
30-E1-I1	occurrence	Non-occurrence
30-E1-I3	occurrence	Non-occurrence

## Conclusions

To examine the field applicability of GGBS concrete mixed with expansive and swelling admixtures, the flow characteristics, strength characteristics, and length variation, of the concrete were monitored and investigated by performing basic performance experiments after selecting the final mix proportions, producing ready-mixed concrete based on the mix proportions, and preparing mock-up test specimens. The conclusions are as follows.

In the basic performance experiments, concrete for which a bentonite-based swelling admixture was used exhibited performance values to similar to those of ordinary Portland cement (OPC) by securing the air content, slump, and compressive strength. However, concrete for which an HPMC-based swelling admixture was used did not exhibit similar performance values. Therefore, concrete with a bentonite-based swelling admixture was chosen as the subject for the mock-up test.
The slump and air content characteristics in each mock-up test specimen were similar to those of the 30-OPC specimen, indicating no influence on fluidity. At 28 d, 30-E1-I1 and 30-E1-I3 developed strengths that were 18.6% and 16.2% higher than that of 30-OPC. Thus, the mix conditions of 30-E1-I1 were practically effective.
(3) 30-E1-I1 exhibited the lowest change in length, followed by 30-E1-I3 and 30-OPC. In the case of 30-OPC, shrinkage cracks occurred continuously over time. Compared to 30-OPC, the drying shrinkage was reduced by 25–50% for 30-E1-I1 and 15–25% for 30-E1-I3. This was attributed to the increase in the content of calcium phosphate, which decreased the expansion effect by hindering the hydration reaction caused by the generation of ettringite and calcium hydroxide by the expansive admixture. Thus, the amount of calcium phosphate must be properly controlled.
When crack monitoring was performed for the mock-up test specimens, 30-OPC had a crack after a certain age with a width of 0.3–0.7 mm and length of 7.0 m. 30-E1-I1 exhibited no cracks, whereas 30-E1-I3 had a crack with a width of 0.05–0.15 mm and length of 0.5 m. Therefore, 30-E1-I1 mix with no crack is the most favorable for field applications.

The above results indicate that GGBS concrete mixed with expansive and swelling admixtures secures the required physical properties depending on the mix design and is expected to be used to produce ready-mixed concrete in various construction works, such as underground parking lots. Continuous research is required on the stable, mid- to long-term, and economic crack reduction.

## Data Availability

All data generated or analyzed during this study are included in this published article.
